# Molecular hydrogen: a preventive and therapeutic medical gas for various diseases

**DOI:** 10.18632/oncotarget.21130

**Published:** 2017-09-21

**Authors:** Li Ge, Ming Yang, Na-Na Yang, Xin-Xin Yin, Wen-Gang Song

**Affiliations:** ^1^ Department of Histology and Embryology, School of Basic Medical Sciences, Taishan Medical University, Tai-an City 271000, Shandong Province, PR China; ^2^ Department of Clinical Medicine, Taishan Medical University, Tai-an City 271000, Shandong Province, PR China; ^3^ Key Laboratory of Atherosclerosis in Universities of Shandong, Taishan Medical University, Institute of Atherosclerosis, Taishan Medical University, Tai-an City 271000, Shandong Province, PR China; ^4^ Department of medical immunology, School of Basic Medical Sciences, Taishan Medical University, Tai-an City 271000, Shandong Province, PR China

**Keywords:** molecular hydrogen, selective anti-oxidation, gaseous signal modulator, preventive and therapeutic applications

## Abstract

Since the 2007 discovery that molecular hydrogen (H_2_) has selective antioxidant properties, multiple studies have shown that H_2_ has beneficial effects in diverse animal models and human disease. This review discusses H_2_ biological effects and potential mechanisms of action in various diseases, including metabolic syndrome, organ injury, and cancer; describes effective H_2_ delivery approaches; and summarizes recent progress toward H_2_ applications in human medicine. We also discuss remaining questions in H_2_ therapy, and conclude with an appeal for a greater role for H_2_ in the prevention and treatment of human ailments that are currently major global health burdens. This review makes a case for supporting hydrogen medicine in human disease prevention and therapy.

## INTRODUCTION

Oxidative stress in the cell results from the robust oxidizing potential of excess reactive oxygen species (ROS) [1]. Acute oxidative stress may result from various conditions, such as vigorous exercise, inflammation, ischemia and reperfusion (I/R) injury, surgical bleeding, and tissue transplantation [2–4]. Chronic/persistent oxidative stress is closely related to the pathogenesis of many lifestyle-related diseases, aging, and cancer [5–8]. However, many clinically tested antioxidants exhibit high toxicity levels that limit their usage to a narrow range of therapeutic dosages, and result in ineffective prevention of oxidative stress-related diseases [9]. Thus, identifying effective antioxidants with little-to-no side effects is very important for the treatment of multiple diseases.

H_2_ is a flammable, colorless, odorless gas that can act as a reducing agent under certain circumstances. It was previously considered physiologically inert in mammalian cells, and was not thought to react with active substrates in biological systems. Recently, H_2_ has emerged as a novel medical gas with potentially broad applications. Dole, *et al*. first reported the therapeutic effects of H_2_ in 1975 in a skin squamous carcinoma mouse model [10]. Thereafter, inhaling high pressure H_2_ was demonstrated as a treatment for liver parasite infection-induced hepatitis [11]. In 2007, Ohsawa and colleagues discovered that H_2_ has antioxidant properties that protect the brain against I/R injury and stroke by selectively neutralizing hydroxyl radicals (·OH) and peroxynitrite(ONOO^-^) [1].

To date, H_2_ preventive and therapeutic effects have been observed in various organs, including the brain, heart, pancreas, lung, and liver. H_2_ mediates oxidative stress and may exhibit anti-inflammatory and anti-apoptotic effects [12–14]. H_2_ not only provides a safe and effective disease treatment mechanism, but also prompts researchers to re-visit the significance and benefits of medicinal gas in the human body. This review summarizes recent progress toward potential preventive and therapeutic applications of H_2_ and addresses possible underlying molecular mechanisms.

## POTENTIAL MECHANISMS OF H_2_ AS A THERAPEUTIC AGENT

**Figure 1:**
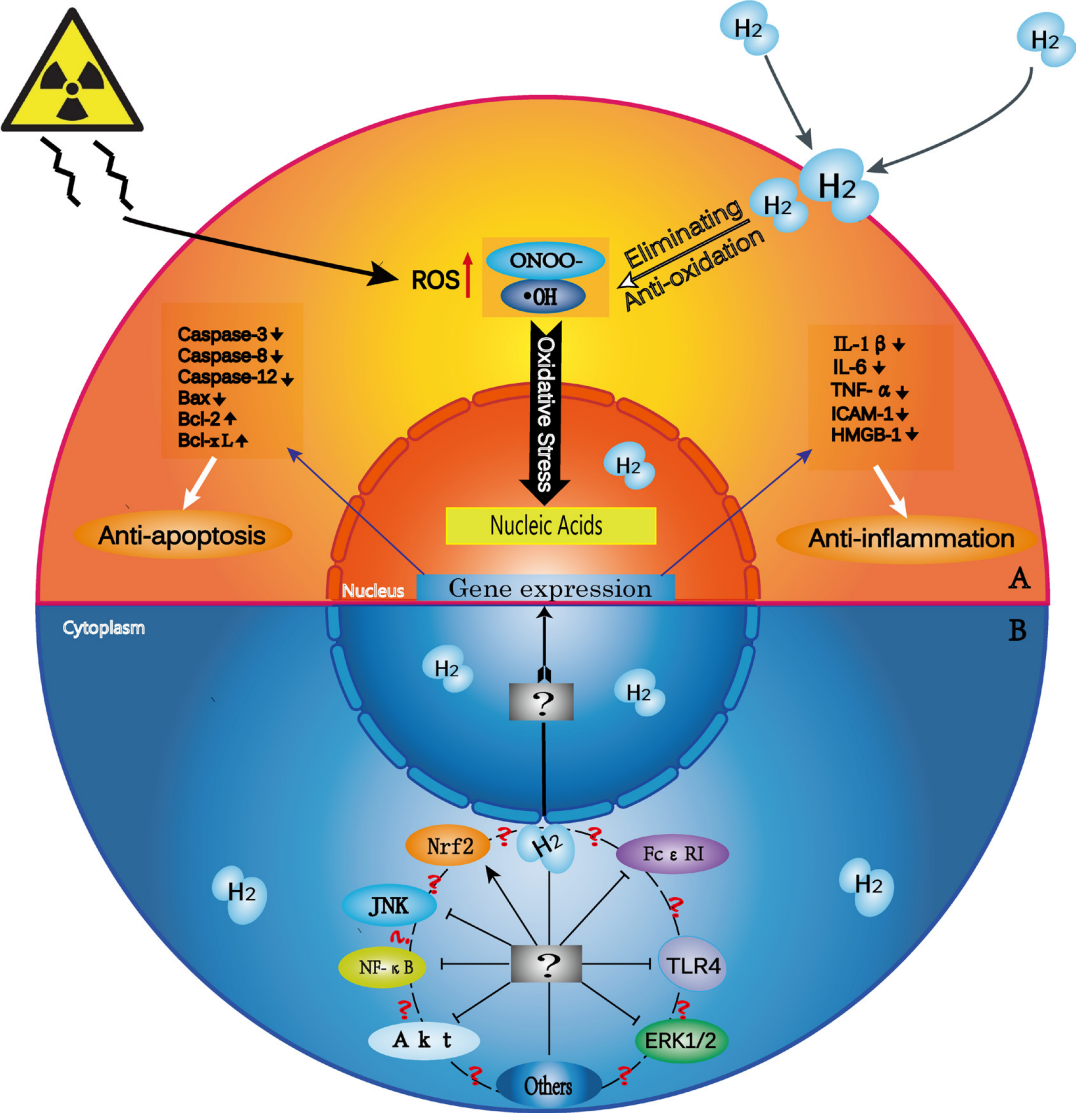
H_2_ biological effects and possible mechanisms of action (**A**) H_2_ has selective anti-oxidative, anti-inflammatory and anti-apoptotic properties. Exogenous damage due to such factors as radiation induces excess cellular ROS production. H_2_ penetrates biomembranes and effectively reaches cell nuclei. H_2_ selectively scavenges ·OH and ONOO- and thus prevents DNA damage. H_2_ also downregulates the expression of pro-inflammatory and inflammatory cytokines, such as IL-1β, IL-6, TNF-α, ICAM-1, and HMGB-1, and of pro-apoptotic factors, such as caspase-3, caspase-12, caspase-8 and Bax. H_2_ upregulates the expression of anti-apoptotic factors, such as Bcl-2 and Bcl-xL. (**B**) H_2_ modulates signal transduction within and between many pathways. ?¶The exact targets and molecular mechanisms of H_2_ are unknown. ?: Does cross-talk occur among various signaling pathways? If so, how is it triggered? Further studies should explore other signaling pathways that may take part in H_2_-related disease mitigation.

The exact molecular mechanisms of the effects of low-dose H_2_ remain unclear. H_2_ can modulate signal transduction across multiple pathways, but its primary molecular targets have not been determined. Examining critical overlapping signaling molecules would help mapcrosstalk among critical pathways. To fully explain the biological functions of H_2_, its molecular mechanisms of action must be clarified. Potential mechanisms are proposed and summarized in Figure 1.

### Selective anti-oxidation

The role of H_2_ as an antioxidant has garnered the most attention among many proposed biological activities. H_2_ is a specific scavenger of ·OH and ONOO-, which are very strong oxidants that react indiscriminately with nucleic acids, lipids, and proteins, resulting in DNA fragmentation, lipid peroxidation, and protein inactivation. Fortunately, H_2_ does not appear to react with other ROS that have normal physiological functions *in vivo* [1].

H_2_ administration decreases expression of various oxidative stress markers, such as myeloperoxidase, malondialdehyde, 8-hydroxy-desoxyguanosine8-OHdG, 8-iso-prostaglandin F2a, and thiobarbituric acid reactive substances in all human diseases and rodent models [15–19]. Recent reports also revealed that H_2_-selective anti-oxidation mitigates certain pathological processes in plants and retains freshness in fruits [20–23]. In 2016, researchers proposed that H_2_ could decrease ROS content in *Ganoderma lucidum* depending on the presence of endogenous glutathione peroxidase [24].

### Anti-inflammation

A 2001 study found that breathing high-pressure H_2_ could cure parasite-induced liver inflammation, and was the first demonstration of the anti-inflammatory properties of H_2_ [11]. H_2_ has exhibited anti-inflammatory activities in various injury models. Typically, H_2_ inhibits oxidative stress-induced inflammatory tissue injury via downregulation of pro-inflammatory and inflammatory cytokines, such as interleukin (IL)-1β, IL-6, tumor necrosis factor-α(TNF-α) [25, 26], intercellular cell adhesion molecule-1 [27], high-mobility group box 1(HMGB-1) [27], nuclear factor kappa B (NF-κB) [28], and prostaglandin E_2_ [29]. H_2_ improved survival rate and reduced organ damage inseptic mice by downregulating early and late pro-inflammatory cytokines in serum and tissues, suggesting the potential use of H_2_ as a therapeutic agent for conditions associated with inflammation-related sepsis/multiple organ dysfunction syndrome [30]. Additionally, H_2_ released from intestinal bacteria has been suggested to suppress inflammation [31].

### Anti-apoptosis

H_2_ exerts anti-apoptotic effects by up- or downregulating apoptosis-related factors. For example, H_2_ inhibits expression of the pro-apoptotic factors, B-cell lymphoma-2-associated X-protein [32], caspase-3 [33], caspase-8 [32], and caspase-12 [34], and upregulates the anti-apoptotic factors, B-cell lymphoma-2 and B-cell lymphoma-extra large [32, 35]. H_2_ further inhibits apoptosis by regulating signal transduction within and between specific pathways. Hong, *et al.* first confirmed in 2014 that the H_2_-triggered neuroprotective effect is at least partially associated with anti-apoptotic protein kinase B pathway (also known as the Akt/glycogen synthase kinase 3β(GSK3β) pathway)activation in neurons [35].

### Gene expression alterations

H_2_ administration induces expression of diverse genes, including NF-κB [36], c-Jun N-terminal kinase (JNK) [37, 38], proliferation cell nuclear antigen [39], vascular endothelial growth factor (VEGF) [40], glial fibrillary acidic protein (GFAP) [41, 42], and creatine kinase [43]. Some of these molecules may be secondarily regulated by H_2_, and some may be direct H_2_ targets. In the normal rat liver, H_2_ was found to have little effect on the expression of individual genes, but gene ontology analysis demonstrated upregulation of oxidoreduction-related genes [44]. The anti-inflammatory and anti-apoptotic properties of H_2_ could be realized by modulating expression of pro-inflammatory and inflammatory cytokines, and apoptosis-related factors.

### H_2_ as a gaseous signal modulator

Oxidative stress impacts multiple signaling pathways, including the extracellular signal-regulated protein kinase (ERK)1/2, NF-κB, JNK, andnuclear factor-erythroid 2p45-related factor 2 (Nrf2) pathways. Along with selectively scavenging ·OH, H_2_ may alleviate oxidative stress-induced injury by targeting these pathways [45–47]. Additional studies confirmed that H_2_ could exert anti-inflammatory effects by regulating Toll-like receptor 4 (TLR4) signaling [48], and anti-apoptotic effects through Ras-ERK1/2-MEK1/2 and Akt pathway inactivation [49]. H_2_ may also protect against allergic reactions by directly modulating FcεRI-related signaling, rather than through radical-scavenging activity [50].

Since H_2_ may influence multiple signaling pathways to exert broad effects, crosstalk between these pathways likely influences H_2_ therapeutic outcomes. The effects of H_2_ as a gaseous signal modulator in a therapeutic setting may involve a network of signaling molecules, and future research using various animal and cell models is needed to confirm the benefits of H_2_ in such settings.

## H_2_ DELIVERY MECHANISMS

### Inhalation

Researchers have explored several convenient and effective delivery systems for H_2_ administration *in vivo* (Table 1). A simple method of administering H_2_ therapeutically is by inhalation using a ventilator circuit, facemask, or nasal cannula. Patients typically inhale H_2_ through a facemask, whereas in animal models, H_2_ is commonly administered through a ventilator that provides H_2_ electrolyzed from water. Inhaled H_2_ acts rapidly and may be used to treat acute oxidative stress [51]. An experiment in rats showed that inhalation of H_2_ mixed with nitrous oxide, O_2_, and N_2_ dose-dependently increased levels of H_2_ dissolved in arterial blood to higher concentrations than in venous blood, demonstrating that administered H_2_ was incorporated into tissues [1]. H_2_ inhalation caused no observable adverse effects and had no effects on blood pressure [1] or other blood parameters, such temperature, pH, and pO_2_ [52]. H_2_ inhalation was safe and effective in patients with acute cerebral infarction [53]. Recent findings suggest that H_2_ treatment is neuroprotective in patients with cerebral I/R injury [54]. H_2_ also mitigates surgery-induced cognitive impairment [55], decreases lung graft injury [56] and radiation-induced skin injury in rats [57], and attenuates lipopolysaccharide-induced acute lung injury in mice [14].

**Table 1:** *In vivo* H_2_ delivery systems

Administration	Preparation/delivery method	Characteristics
Inhalation	Inhale gas mixture containing H_2_ (< 4%) [1, 52–53]	1. Rapid action, straightforward delivery, but unsafe. 2. Does not influence blood physiological parameters (temperature, blood pressure, pH, pO_2_). 3. Suitable to defense against acute oxidative stress. 4. Unpractical to dose continuously.
Oral intake of hydrogen-rich water (HW)	Dissolving H_2_ in water up to 0.8 mM under atmospheric pressure at room temperature. Drinking HW [58, 63]	1. Convenient, easily administered, safe, efficient. 2. Easily evaporates and is lost in the stomach or intestine. 3. Difficult to control H_2_ concentration administered.
Injection of hydrogen-rich saline(HS)	Intravenous injection[122]	Delivery of more accurate H_2_concentrations.
	Intraperitoneal injection[25]	
	Intrathecal injection [68]	
	Intravitreal injection [201]	
Direct incorporation	Bath [69]	1. Low cost. 2. Convenient and safe.
	Cold storage of transplanted organs [71]	
	Eye drops [72]	
	Spray on plants or immerse plants [22]	
Increased intestinal hydrogen	Oral drugs (e.g. acarbose, lactulose)[88]	1. Low cost. 2. Convenient.
	Dietary(e.g. turmeric) [86]	

### Oral intake of hydrogen-rich water

While inhalation of H_2_ produces rapid effects, this delivery method may not be practical for daily preventive therapy. Due to safety concerns, H_2_ concentrations and dosages must be strictly controlled. Unlike gaseous H_2_, solubilized H_2_ [H_2_-dissolved water or hydrogen-rich water (HW)] is portable, safe, and easily administered [58]. H_2_ can be dissolved in water up to 0.8 mM (1.6 mg/L) under atmospheric pressure at room temperature without changing pH, and 0.8 mM HW effectively improved obesity in mice model [59]. Additionally, H_2_ accumulation in the liver after oral HW administration can be measured with a needle-type hydrogen electrode to determine whether consumption of small amounts of H_2_ over a short time period can efficiently improve various disease models. *In vitro* experiments demonstrated that carbohydrate polymers, including glycogen and starch, have an affinity for H_2_ [60], and some studies found that drinking HW produced beneficial effects in disease models, such as Parkinson's disease [61], oral palatal wound [62], radiation-induced oxidative injuries [63], periodontal tissue aging [64], and depressive-like behavior [65].

### Injection of hydrogen-rich saline

Although administering oral HW is safe and convenient, controlling the concentration of H_2_ administered can be difficult, as it evaporates in water over time and can be lost before absorption in the gastrointestinal tract. Thus, hydrogen-rich saline (HS) injections may deliver more accurate H_2_ doses [66]. Experimental evidence suggests that HS could be successfully administered by peritoneal or intravenous injection. For example, HS injection had neuroprotective effects in a spinal cord injury rat model [41]. HS treatment could also be used as an effective radioprotective agent through free radical scavenging [67], and improved survival and neurological outcome after subarachnoid hemorrhage (SAH) [25]. Additionally, intrathecal injection of HS produced analgesic effects in neuropathic rats by reducing activation of spinal astrocytes and microglia [68].

### Direct diffusion of hydrogen: baths, eye drops, and immersion

Because H_2_ can easily penetrate the skin and be distributed via blood flow throughout the body, a warm HW bath can be used therapeutically in daily life. Warm HW baths may minimize UVA-induced skin damage [69]. A cold storage device equipped with a HW bath may be cytoprotective in various diseases and in organ transplantation. In 2011, Buchholz, *et al.* demonstrated that storage of intestinal grafts in a preservation solution containing high levels of H_2_ prevented graft damage after reperfusion [70]. In 2013, Noda, *et al*. found that H_2_ delivery to cardiac grafts during cold preservation efficiently ameliorated myocardial injury due to cold I/R. This new method for saturating organs with H_2_ during cold storage should be further developed for potential therapeutic and preventative use during transplantation [71].

H_2_ dissolved in saline has also been used to directly treat the ocular surface [72, 73]. Direct application of eye drops containing H_2_ ameliorated I/R injury of the retina in a rat model [72]. Antioxidant therapy via an H_2_-enriched irrigation solution has been suggested as a new potent corneal treatment to prevent blindness caused by alkali burn [73].

HW immersion has also drawn recent widespread attention in plant physiology. H_2_ was preliminarily suggested to act as a novel bioregulator involved in phytohormone signaling [74], root development [22, 75], delay of fruit senescence [23], and plant responses to various stressors, including paraquat [76], ultraviolet radiation [77, 78], drought [79], salinity [80], and cadmium [81], aluminum (Al) [21], and mercury exposure [20, 21].

### Increased intestinal hydrogen

H_2_ is spontaneously produced in the body through fermentation of undigested carbohydrates by resident enterobacterial flora [82]. *Escherichia coli* can produce a considerable amount of H_2_ through the hydrogenase enzyme. However, few groups have studied the physiological and therapeutic functions of H_2_ derived from the gastrointestinal tract. H_2_ produced by bacterial fermentation in the gut shortens colonic transit, and this effect was more prominent in the proximal than the distal colon [83]. Kawai, *et al.* demonstrated that H_2_ released from intestinally colonized bacteria could alleviate concanavalin A-induced mouse hepatitis [31]. Endogenous H_2_ also mediated the suppression of colon inflammation induced by dextran sodium sulfate [84].

Recent work suggests that some oral drugs and foods stimulate intestinal H_2_ production, and these findings may expand the role of H_2_ in disease treatment. Acarbose, an α-glucosidase inhibitor, increased H_2_ production and neutralized oxidative stress in the gastrointestinal tract. Thus, Suzuki, *et al.* proposed that H_2_ produced by intestinal bacteria acts as a unique antioxidant and prevents cardiovascular events [85]. Dietary turmeric also induced H_2_ production by intestinal bacteria [86], and lactulose was shown to be an indirect antioxidant ameliorating inflammatory bowel disease [87, 88]. These examples illustrate that endogenous H_2_ production has important consequences in the human body.

## PREVENTIVE AND THERAPEUTIC APPLICATIONS OF H_2_

Safety is a primary concern with respect to H_2_ transportation, storage, and administration. H_2_ is flammable only at temperatures greater than 527°C, and explodes by rapid chain reaction with oxygen in the H_2_ concentration range of 4–75% (vol/vol) [89, 90]. As H_2_ is not cytotoxic even at high concentrations, high-pressure H_2_ has been safely used in deep-diving gas mixes to prevent decompression sickness and arterial gas thrombi [91–93]. Because inhaling 1–4% H_2_ has demonstrated great efficacy in medical applications, the use of H_2_ at such low concentrations has been deemed feasible and safe [1, 94].

**Figure 2:**
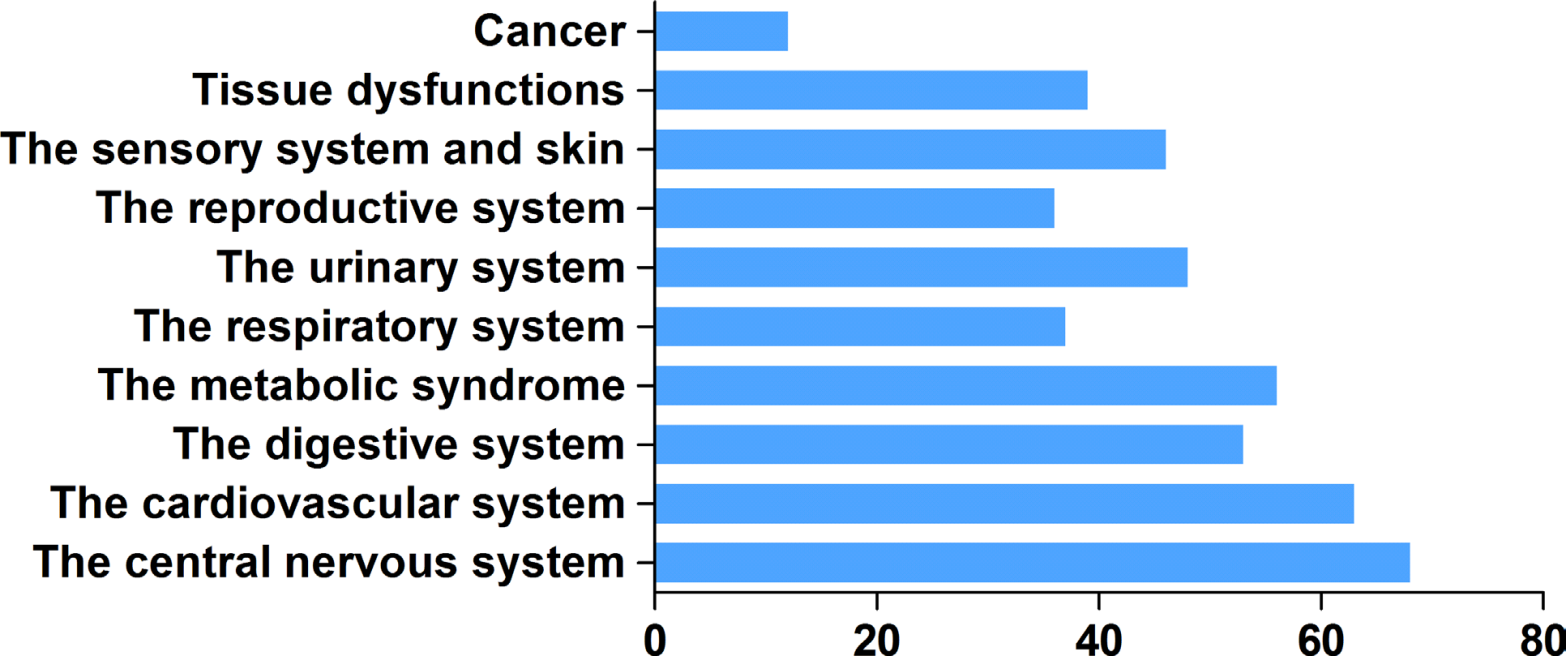
Number of publications on H_2_ biological effects in various organ system diseases since 2007

H_2_ has unique advantages in clinical applications. It effectively penetrates biomembranes to reach cell nuclei and mitochondria [90], and can easily penetrates the blood–brain barrier by gaseous diffusion, while most antioxidant compounds cannot. Real-time monitoring of H_2_ diffusion can be accomplished by measuring H_2_ concentrations inside various tissues using electrodes [72, 94]. As of March 2017, the number of publications on the biologically or medically beneficial effects of H_2_ had surpassed 450 (Figure 2). H_2_ administration has shown preventive and therapeutic effects in a wide range of disease models and human diseases (Supplementary Table 1). Thus, this review will summarize the results of recent experimental and clinical examinations of actual H_2_ applications.

### Effects of hydrogen on central nervous system diseases

Because H_2_ can penetrate the blood–brain barrier by gaseous diffusion [1, 95], the therapeutic effects of H_2_ on central nervous system diseases have been studied extensively. Ohsawa and colleagues reported in 2007 that inhaled H_2_ reduced infarct size in a focal cerebral I/R injury rat model [1]. Parkinson's disease researchers found that oral HW, even at concentrations as low as 5%, alleviated symptoms in murine models by reducing oxidative stress [61, 96]. Further study indicated that drinking HW and intermittent H_2_ exposure were more effective than continuous H_2_ exposure [97]. Yoritaka, *et al.* recently demonstrated that drinking HW reduced oxidative stress and improved patient symptoms in a Parkinson's disease clinical trial [98]. Moreover, endogenous H_2_ maybe closely related to the pathogenesis of Parkinson's disease. Brenner, *et al.* found that environmental toxins deteriorated intrinsic melanin, and that melanin could split the water molecule into hydrogen and oxygen, suggesting that a lack of endogenous H_2_ could accelerate Parkinson's disease processes [99]. H_2_ has also been studied as a potential treatment for Alzheimer's disease, another neurodegenerative condition. Li, *et al.* reported that HS injection improved cognitive and memory functions in an Alzheimer’s-like rat model by preventing neuroinflammation and oxidative stress [100], likely due in part to H_2_-mediated suppression of abnormal IL-1β, JNK, and NF-κB activation [47].

In addition to neurodegenerative diseases, H_2_ administration also appears to alleviate other brain diseases and injuries, such as hypoxia-ischemia (HI) brain injury [101], stress or age-related cognitive impairment [95, 102], traumatic brain injury [103], cerebral I/R injury [104–106], and SAH-induced early brain injury [25, 107] in rodent models. However, conflicting observations have been made regarding the effects of H_2_ on rat brain damage. Some researchers reported beneficial effects of H_2_ therapy in the neonatal HI rat model [66], while others considered H_2_ ineffective [108]. These opposing findings might be due to differing experimental conditions, such as different degrees of HI insult, age of pups, H_2_ concentration, and length of H_2_ exposure. A recent study showed that H_2_ administration without surgery did not exert neuroprotective effects or improve functional outcomes in rats after intracerebral hemorrhage [109]. For spinal cord injury, H_2_ treatment improved locomotor behavior recovery in rats [110] and neurological recovery in mice with experimentally-induced autoimmune encephalomyelitis [111].

### Effects of hydrogen on cardiovascular system diseases

Evidence suggests that H_2_ treatment protects against myocardial injury and development of atherosclerosis and other vascular diseases. H_2_ inhalation limited myocardial infarction extent without altering hemodynamic parameters in a rat model of myocardial I/R injury [94], consistent with other reports that HS injection provided cardioprotection against I/R injury [112–115]. Myocardial cold I/R injury following heart transplantation is a major determinant of primary graft dysfunction and chronic rejection [116], and can promote the subsequent development of graft coronary artery disease [117]. Researchers found that H_2_ inhalation ameliorated rat cardiac cold I/R injury [118], and drinking HW daily may protect cardiac and aortic allograft recipients from inflammation-associated deterioration [119]. Noda, *et al.* recently established a novel method of preserving cardiac grafts using a HW bath [71]. Soluble H_2_ delivered to excised cardiac grafts during cold preservation ameliorated cold I/R injury in grafts from syngeneic older donors and in allografts subjected to extended cold storage [71].

In addition to treating myocardial I/R injury, HS treatment prevented left ventricular hypertrophy in spontaneously hypertensive rats [120], isoproterenol-induced rat myocardial infarction [113], and doxorubicin-induced rat myocardial injury [121], and improved survival and neurological outcomes after cardiac arrest/resuscitation in rats [122]. Drinking HW alleviated radiation-induced myocardial injury in mice [123]. H_2_ inhalation also improved survival and functional outcomes in a post-cardiac arrest syndrome rat model [124]. In 2008, Ohsawa, *et al.* found that oral HW prevented atherosclerosis development in anapolipoprotein E knockout mouse model [125]. HS administration has been shown to prevent neointima formation after carotid balloon injury by suppressing ROS and the TNF-α/NF-κB pathway [126], as well as cerebral vasospasm occurrence after SAH by limiting vascular inflammation and oxidative stress in rats [127].

### Effects of hydrogen on digestive system diseases

In 2001, Gharib, *et al.* discovered that breathing high-pressure H_2_was protective against parasite-induced liver injury [11]. Subsequent studies demonstrated HW therapeutic effects in concanavalin A-induced mouse hepatitis [31] and chronic hepatitis B in patients [128]. Liver fibrosis is a universal consequence of chronic liver diseases, and sustained hepatocyte injury initiates an inflammatory response. H_2_-mediated suppression of liver fibrogenesis in mice may be mediated mainly by ·OH scavenging, which protects hepatocytes from injury [58]. In a cirrhotic rat model, HS combined with N-acetylcysteine alleviate doxidative stress and angiogenesis [40]. H_2_ inhalation also reportedly protects against hepatic I/R injury [129]. Liu, *et al.* demonstrated that intraperitoneal injection of HS might be a widely applicable method to attenuate hepatic I/R injury in a rat model [130]. Additionally, many studies have demonstrated protective effects of H_2_ in other liver diseases, such as radiation-induced damage in liver tumor patients [131], acetaminophen-induced hepatotoxicity [132], obstructive jaundice-induced liver damage [45, 133], nonalcoholic steatohepatitis and hepatocarcinogenesis [134], postoperative liver failure after major hepatectomy [135], liver regeneration after partial hepatectomy [39], and acute hepatic injury in acute necrotizing pancreatitis [136] in murine models. Recent work confirmed that HS improved nonalcoholic fatty liver disease by alleviating oxidative stress and activating peroxisome proliferatoractivated receptor α (PPARα) and PPARγ expression in rat hepatocytes [137].

Intestinal I/R injury occurs in a variety of clinical settings, such as surgical treatment for abdominal aortic aneurysm, small intestinal transplantation and mesenteric artery occlusion. Inflammation and oxidative stress induced by intestinal I/R injury are the primary causes of surgical treatment [138, 139]. Injection of HS/hydrogen-rich solution reduced inflammation and oxidative stress in an I/R injury rat model, and was protective against intestinal contractile dysfunction and damage [140–142]. Poor preservation and I/R injury during small intestinal transplantation are still major causes of recipient morbidity and mortality. Buchholz, *et al.* demonstrated in 2008 that H_2_ treatment ameliorated transplant-induced intestinal injuries, including mucosal erosion and mucosal barrier breakdown, in a rat small intestinal transplant model [27]. Three years later, the same group demonstrated that intestinal grafts preloaded with H_2_ exhibited superior morphology and function in rodent intestinal transplants, ultimately facilitating recipient survival [70]. HS treatment also alleviated colonic mucosal damage [143] and postoperative ileus [144] in murine models.

H_2_ administration also has been shown to effectively treat stress-associated gastric mucosa damage [145] and aspirin-induced gastric lesions [146]. Xue, *et al.* found that drinking hydrogen-rich electrolyzed water suppressed the dose-response effect of aspirin-induced gastric injury in a rat model [147]. HS injection also reduced the severity of acute pancreatitis [13, 28] and I/R injury after pancreatic transplantation in rats [148].

### Effects of hydrogen on metabolic syndrome

Metabolic syndrome refers to a common disorder characterized by a combination of obesity, dyslipidemia, hypertension, and insulin resistance [149]. Oxidative stress has been implicated in metabolic syndrome [150], and many studies have demonstrated protective effects of H_2_ in metabolic disorders [19, 151–153]. In some specific metabolic syndrome rat models, colonic H_2_ generated from fructan appeared to mitigate inflammation-induced oxidative stress [151]. HW also prevented glomerulosclerosis and ameliorated creatinine clearance [153]. Moreover, HS administration decreased plasma low-density lipoprotein cholesterol (LDL-C) levels and improved high-density lipoprotein (HDL) function in hamsters fed a high fat diet [154]. For patients with potential metabolic syndrome, HW consumption downregulated oxidative stress indicators and enhanced superoxide dismutase (SOD) levels, thereby increasing endogenous antioxidant defense against O2–· [19]. HW consumption also decreased patient serum LDL-C levels and improved HDL function [152].

H_2_ treatment has shown positive effects on energy metabolism. Kamimura, *et al.* found that long-term HW consumption decreased body fat and weight, along with plasma glucose, insulin, and triglyceride levels, by stimulating energy metabolism [59]. This work found that H_2_ treatment increased expression of the hepatic hormone, fibroblast growth factor 21, which enhances fatty acid and glucose expenditure [59].

H_2_ treatment also mitigates type-2 diabetes development by reducing oxidative stress and improving glucose metabolism [155]. Based on the observation that acarbose induces endogenous H_2_ production, Suzuki, *et al.* discovered that acarbose treatment increased exhaled H_2_ concentrations, reducing the risk of cardiovascular disease in patients with impaired glucose tolerance or type-2 diabetes. These benefits can be attributed, at least in part, to the ability of acarbose to neutralize oxidative stress by increasing H_2_ production in the gastrointestinal tract [85]. Amitani, *et al.* demonstrated that H_2_ could exert metabolic effects similar to those of insulin and may also be a novel therapeutic alternative to insulin in the treatment of type 1 diabetes mellitus [156].

### Effects of hydrogen on respiratory system diseases

H_2_ treatment is beneficial in treating diverse respiratory system diseases. HS injection is protective against acute pulmonary I/R injury in rat [157] and rabbit [158] models via anti-oxidative, anti-inflammatory, and anti-apoptotic mechanisms. H_2_ inhalation also ameliorated lung transplant-induced I/R injury [32, 159]. Meng and colleagues recently demonstrated that inflation with CO or H_2_ protected against I/R injury in a rat lung transplantation model, and this effect was enhanced by combined CO and H_2_ treatment. H_2_ might exert protective effects through CO regulation, which could explain why the combination treatment exhibited greater protective effects. However, this study did not measure CO and H_2_ concentrations in recipient blood, and optimal CO and H_2_ concentrations must be further explored [160].

Recent studies have focused on H_2_ protection against sepsis-related lung injury. HS treatment inhibited sepsis-induced acute pulmonary injury in rats, possibly as a result of HS anti-oxidative and anti-inflammatory activities [161]. H_2_ inhalation also protected against sepsis-related lung injury by reducing inflammatory cytokine HMGB1 levels in septic mice, and this was partially mediated through activation of hemeoxygenase 1(HO-1) and its upstream regulator, Nrf2 [162]. In 2016, Tao, *et al.* demonstrated that HS administration preserved levels of aquaporin 1 (AQP1) and AQP5, which eliminate extravascular lung water, to alleviate sepsis-related lung injury by inhibiting p38 mitogen-activated protein kinase and JNK activation [37]. These observations provide potential new therapeutic targets for sepsis-related lung injury.

Studies have also shown that H_2_ improves lung injuries induced by many other factors, such as hyperoxia [163, 164], lipopolysaccharides [14, 17], smoke inhalation [165], paraquat[166], monocrotaline [167], and extensive burns [168]. A 2013 study showed that HS pretreatment ameliorated cigarette smoking-induced airway mucus production and airway epithelium damage in rats [169]. Xiao, *et al.* found that HS reduced airway inflammation and remodeling in asthmatic mice via NF-κB inactivation [46].

### Effects of hydrogen on urinary system diseases

Renal I/R injury, an important cause of acute kidney injury, is unavoidable during various clinical situations, such as renal transplantation, partial nephrectomy, and treatment of suprarenal aortic aneurysms [170–172]. The mechanisms responsible for renal damage remain largely unknown, although ROS, inflammatory responses, and apoptosis are likely involved [173, 174]. Recent findings suggest that H_2_ protects against renal I/R injury, mainly due to H_2_ anti-inflammation and anti-apoptosis effects and selective reduction of cytotoxic ROS [175, 176].

Abe and colleagues associated I/R-induced acute renal injury with decreased allograft survival in patients with transplanted kidneys [177]. Allograft pre-preservation in Hydrogen-rich University of Wisconsin(HRUW) solution attenuated renal cold I/R injury caused by renal transplantation, and suppressed cytotoxic ROS generation, renal tubular injury, and interstitial fibrosis, leading to superior long-term renal graft outcomes [177]. Pre-preservation had no effect on interferon-γ, IL-6, and TNF-α expression. A 2010 study demonstrated that oral administration of HW attenuated local production of these inflammatory markers in a kidney allotransplantation setting [178]. We attribute differences in these findings to diverse H_2_ delivery systems and durations, and we suggest that long-term oral administration of HW appeared to have better therapeutic effects than transient pre-preservation in HRUW. Recent work indicates that HS protects against acute renal injury after liver transplantation partly by reducing apoptosis, which was possibly involved in modulating p53-mediated autophagy [33].

Various animal models have been established to study the therapeutic effects of H_2_ on renal injury. Nakashima-Kamimura, *et al.* reported in 2009 that both H_2_ inhalation and oral HW alleviated cisplatin-induced nephrotoxicity without compromising anti-tumor activity [60]. More recent evidence indicated that H_2_ alleviates renal injury induced by many factors, such as ferric nitrilotriacetate-induced nephrotoxicity [179], glucose and α, β-dicarbonyl compound-induced oxidative stress [180], unilateral ureteral obstruction [181], spontaneous hypertension [36], glycerol [43], septic shock [182], acute pancreatitis [183], and burns [184].

At present, few groups have published studies on the effects of H_2_ in the bladder. Matsumot, *et al.* found no obvious efficacy of HW in patients with interstitial cystitis/painful bladder syndrome, although supplementation with HW effectively relieved bladder pain in some cases [185]. Appropriately designed, large scale, prospective clinical studies will be required to confirm these findings.

### Effects of hydrogen on reproductive system diseases

H_2_ has also been applied in reproductive system ailments, primarily testicular injury. The testis is highly sensitive to damage during therapeutic irradiation[186], and radiotherapy can induce azoospermia or infertility[187]. In 2012, Chuai and colleagues demonstrated that HS attenuated male germ cell loss and protected spermatogenesis with no adverse side effects in a radiation-induced mouse model [188, 189]. This represented the first *in vivo* evidence to suggest H_2_ radioprotection through ·OH neutralization in irradiated tissue. HS was also shown to play a radio-protective role in a gamma ray-induced rat testicular damage model [190]. Thus, H_2_therapy may effectively preserve fertility in males exposed to irradiation. Additionally, HS protects against I/R- and spinal cord hemisection-induced testicular injuries in rat models [191, 192]. Long-term HS treatment alleviated nicotine-induced testicular oxidative stress in a mouse model [193] and was protective against erectile dysfunction in a streptozotocin-induced diabetic rat model [194].

To date, only two articles have reported the therapeutic effects of H_2_ in female reproductive diseases. In 2011, Yang, *et al.* suggested that HS acts protectively in a preeclampsia rat model via effective anti-oxidation [195]. HS also attenuated chemotherapyinduced ovarian injury in a female rat model by suppressing immoderate oxidative stress, which may regulate the Nrf2/antioxidant response element signaling pathway [196]. While these investigations provide some quantitative basis for the possible use of H_2_ as a radio/chemotherapy-protectant, further studies are necessary to determine the exact mechanisms of action.

### Effects of hydrogen on sensory system and skin diseases

Retinal I/R injury exists in various eye diseases, including glaucoma and other ocular vascular disorders [197]. In 2010, Oharazawa, *et al.* found that administration of H_2_-loaded eye drops protected the retina against acute I/R injury by scavenging ·OH, which is a highly effective neuroprotective and anti-oxidative strategy [72]. Intraperitoneal injection of HS and inhaled high-dose H_2_were both found to confer neuroprotection against retinal I/R injury via anti-oxidative, anti-inflammatory, and anti-apoptotic pathways in rat models [198, 199]. Unexpectedly, HS therapy did not inhibit retinal neovascularization in anoxygen-induced retinopathy mouse model [200]. Additional experiments are needed to explore the pathological and biochemical mechanisms underlying these effects.

H_2_ mitigated retinal diseases induced by other factors, such as glutamate-induced excitotoxic injury [201], light-induced damage [16], optic nerve crush [202], and N-methyl-N-nitrosourea (MNU)-induced retinitis pigmentosa [203]in rodent models. H_2_ may also be a new potent treatment for corneal injury caused by alkali burn [73], and has demonstrated protective effects in ear diseases. H_2_ facilitated the recovery of hair cell function and attenuated noise-induced temporary hearing loss by scavenging detrimental ROS formed in the inner ear in mouse and guinea pig models [204–208]. Another recent study suggested that HS attenuates eosinophil activation in a guinea pig model of allergic rhinitis by reducing oxidative stress [209].

The skin is a biological defense barrier for the body, and skin injuries caused directly by radiation energy or indirectly by free radicals results in radiodermatitis in nearly 95% of patients receiving radiation therapy. H_2_ administration protected against γ or X-ray radiation-induced dermatitis [57, 210] and ultraviolet (UV)-induced skin injury[211] in murine models. In 2013, Shin, *et al.* also observed that the application of atomic hydrogen surrounded by water molecules (H(H_2_O)m) may prevent UV-induced human skin injury [212]. H_2_ administration has also shown potential therapeutic effects in acute erythematous skin diseases [213], skin flap I/R injury in rats [214, 215], and psoriatic skin lesions [216]. A recent study found that autophagy played an important role in HS-attenuated post-herpeticneuralgia (PHN) in rats. Thus, HS may attenuate hyperalgesia and inhibit the release of cytokines TNF-α, IL-1β, IL-6 in rats with PHN by activating autophagy [217].

### Effects of hydrogen on tissue dysfunctions

In 2011, Hanaoka, *et al.* demonstrated that H_2_ protected cultured chondrocytes against oxidative stress by selectively reducing ONOO-[218], suggesting that H_2_ could be used to prevent or treat joint diseases. H_2_ reduced disease activity in rheumatoid arthritis patients [219], alleviated microgravity-induced bone loss [220], suppressed periodontitis progression by decreasing gingival oxidative stress [209, 221–223], and prevented steroid-induced osteonecrosis in rabbits [224, 225].

H_2_ may also exert therapeutic effects in hematological system diseases. Allogeneic hematopoietic stem cell transplantation is a potentially curative therapy for many malignant and nonmalignant hematologic diseases. However, acute graft-versus-host disease (aGVHD) is a lethal complication of hematopoietic stem cell transplantation, which limits its application. HS administration protected against lethal aGVHD in a major histocompatibility complex-incompatible mouse bone marrow transplantation model [226] and increased survival rates in a lethal irradiation-induced mouse model [227]. Sepsis is the most common cause of death in intensive care units. Combination therapy with H_2_ and hyperoxia or HS treatment provides enhanced therapeutic efficacy via both anti-oxidative and anti-inflammatory mechanisms, and might be a clinically feasible approach to treat sepsis [228–231]. Other studies indicated that H_2_ administration accelerated recovery in aplastic anemia mice [232], increased blood alkalinity in physically active men [233, 234], inhibited collagen-induced platelet aggregation in healthy humans and rats [235], and elevated serum anti-oxidative function in thoroughbred horses [236].

Additionally, drinking HW improved mitochondrial and inflammatory myopathies in humans [237], ameliorated Duchenne muscular dystrophy in mice [238], reduced glycerol-induced rhabdomyolysis in rats [43], and alleviated muscle fatigue caused by acute exercise in athletes [239]. In 2013, Chen, *et al.* showed that HS attenuated fetal bovine serum-induced vascular smooth muscle cell proliferation and neointimal hyperplasia by inhibiting ROS production and inactivating Ras-ERK1/2-MEK1/2 and Akt signaling. Thus, HS may prevent human restenosis [49]. HS administration was also shown to ameliorate skeletal muscle [240] and myocardial I/R injury in rats [112, 241].

### Effects of hydrogen on cancer

A growing number of studies have found that human tumor cells can produce more ROS than non-transformed cell lines, promoting cancer cell proliferation, DNA synthesis, angiogenesis, invasion, and distal metastasis [242–244]. In light of the powerful ability of H_2_ to scavenge free radicals, H_2_ administration is being increasingly studied as part of anti-cancer therapies in humans and other animals. Dole, *et al.* noted in 1975 that hyperbaric H_2_ therapy caused skin tumor regression in hairless albino mice with squamous cell carcinoma [10]. Recently, platinum nanocolloid-supplemented HW was reported to exert more rapid antioxidant activities and preferentially inhibited human tongue carcinoma cell growth as compared with normal cells [245]. Ionizing radiation can lead to carcinogenesis, and in 2011, Zhao and colleagues first reported that HS injection protected BALB/c mice against radiation-induced thymic lymphoma [246]. Other studies demonstrated that drinking HW prevented progression of nonalcoholic steatohepatitis and accompanying hepatocarcinogenesis in mice by reducing hepatic oxidative stress, inflammation, and apoptosis [134], and protected against ferric nitrilotriacetate-induced nephrotoxicity and early tumor promotional events in rats [179].

H_2_ can also alleviate adverse effects induced by cancer radiotherapy or anti-tumor drugs. Kang, *et al*. suggested that daily consumption of HW could mitigate radiotherapy-induced oxidative stress and improve quality of life after radiation exposure without compromising anti-tumor effects in patients with liver tumors [131]. Similarly, H_2_ administration protected against cisplatin-induced nephrotoxicity [60, 247], and doxorubicin-induced cardiac and hepatic injury [121]. These findings suggest that H_2_ has potential as an anti-cancer therapeutic, and could be used to reduce radio/chemotherapeutic side effects in patients.

### Hydrogen in current clinical healthcare

H_2_ is difficult to dissolve in water, and this initially limited its therapeutic applications. In 2009, Japan solved this technical problem and produced HW. In 2012, HW sales in Japan online alone reached 20 billion yen. In the same year, researchers from 12 developed countries, including the United States and Germany, began developing H_2_ as a healthcare product, and the global HW market reached $22 billion. H_2_ industries continue to grow, and now include H_2_-based hydrogen-rich peripheral products, such as hydrogen health capsules, hydrogen cosmetics, hydrogen-rich bathing agents, and hydrogen ventilator equipment. The first Chinese state-owned HW brand, “Hydrovita,” was established in Beijing in 2013. The Chinese State Drug Administration subsequently defined H_2_ inhalation as medical behavior in 2015. The Chinese H_2_ market will likely be very large, since there are nearly 300 million chronic disease patients in this country. Accordingly, H_2_ products have a promising future as safe, simple, convenient products for health maintenance, with broad potential applications [248].

## FUTURE DIRECTIONS: PROBLEMS TO BE RESOLVED

Although H_2_ has promising preventive and therapeutic applications in various diseases, many problems remain unresolved. Roughly 40 g of carbohydrate is thought to enter the normal human colon each day, so enormous (12,000 ml/day) quantities of H_2_ should be released into the colonic lumen [249–251]. The amount of intestinal H_2_ produced is much larger than that of H_2_ absorbed from water or gas, but only the effects of exogenously administered H_2_ have attracted the attention of the medical field at present. However, intestinal H_2_ also been shown to have beneficial effects in disease remission. In a mouse model, restitution of a hydrogenase-positive *E. coli* strain ameliorated concanavalin A-induced hepatitis [31], although drinking HW was more effective than restitution of hydrogenase-positive bacteria in this study. The fact that some exogenous oral drugs or foods stimulate intestinal H_2_ production supports the development of combination therapies in animal models and clinical trials. We propose that intestinal H_2_ therapies could expand the role of H_2_ in disease treatment.

No H_2_ dose-response effects have been observed thus far. Drinking HW reduced dopaminergic neuron loss in a mouse model of Parkinson's disease. Notably, H_2_ concentrations as low as 0.08 ppm exhibited nearly the same effects as saturated HW (1.5 ppmH_2_) [96]. After HW is consumed, most H_2_ in the blood is undetectable within 30 min [178], likely due to expiration from the lungs. Thus, how a low amount of HW over a short exposure period can be effective remains unknown. However, Kamimura and colleagues found that H_2_ could accumulate in the liver with glycogen, which may partly explain this phenomenon [59]. In another example, as a 2% gas, the amount of H_2_ exposed to a 60-kg person for 24 h would be 104 or more times higher than that administered by drinking saturated HW. Nevertheless, HW is as effective as, and sometimes more effective than, H_2_ [252]. Therefore, the amount of administered H_2_ seems to be independent of the magnitude of effects in many cases.

Additionally, the molecular mechanisms and primary molecular targets of exogenously administered low-dose H_2_ are still unclear. Although H_2_ regulates the expression of various genes and protein activation states, it remains to be determined whether such modulations are the cause or result of the physiological effects of H_2_. Another important question is how H_2_ utilizes and effects crosstalk among anti-oxidative, anti-inflammatory, anti-apoptotic, and other biochemical pathways [89]. Far fewer clinical trials examining H_2_ applications have been conducted compared with the many animal model experiments. Nevertheless, promising applications for H_2_ treatment are expected to emerge for many human diseases, and personalized treatments for patients are a therapeutic goal. Thus, appropriately designed, large-scale, prospective clinical studies are warranted to optimize H_2_ dose, timing, and delivery methods.

## CONCLUSIONS

H_2_ administration is a promising therapeutic option for the treatment of a variety of diseases. This article reviewed current medical research progress with respect to H_2_, including its unique properties, possible mechanisms of action, delivery methods, applications in animal models and clinical trials, and future applications in the field. Although important questions remain unanswered, H_2_-based therapies show great promise as novel and innovative tools to prevent and treat human ailments that are currently major health burdens globally. A better understanding of H_2_ pharmacokinetics and biological mechanisms of action will no doubt advance this important molecule in clinical applications.

## SUPPLEMENTARY MATERIALS FIGURES AND TABLES




