# Supplementary material for: Molecular hydrogen: a preventive and therapeutic medical gas for various diseases
Source: Oncotarget. 2017 Sep 21;8(60):102653–73. doi: 10.18632/oncotarget.21130 (PMC5731988; doi:10.18632/oncotarget.21130)
Supplement: Supplementary file 1 [file oncotarget-08-102653-s001.pdf]

Molecular hydrogen: a preventive and therapeutic medical gas for various diseases

SUPPLEMENTARY MATERIALS

Supplementary Table 1: H_2 therapy-relevant disease models and human diseases. See Supplementary_Table_1
