# Supplementary material for: Molecular hydrogen: a preventive and therapeutic medical gas for various diseases
Source: Oncotarget. 2017 Sep 21;8(60):102653–73. doi: 10.18632/oncotarget.21130 (PMC5731988; doi:10.18632/oncotarget.21130)
Supplement: Supplementary file 2 [file oncotarget-08-102653-s002.docx]

**Supplementary Table 1: H_2_ therapy-relevant disease models and human diseases**

| **Disease/pathological and physiological condition** | **Species** | **References** |
| --- | --- | --- |
| **Central nervous system** |  |  |
| Parkinson’s disease | Rat | [61,97] |
|  | Mouse | [96] |
|  | Human | [98] |
| Alzheimer’s disease | Rat | [47, 100] |
| Stress-induced cognitive impairments in hippocampus-dependent learning tasks | Mouse | [95] |
| Hippocampus endoplasmic reticulum stress  Cognitive impairment | Rat  Mouse | [106]  [102] |
| Traumatic brain injury | Rat | [103] |
| Global cerebral I/R injury | Rat | [104-105] |
| Hemorrhage-induced early brain injury | Rat | [25] |
| Subarachnoid hemorrhage | Rabbit | [107] |
| Neonatal hypoxia-ischemia brain damage | Rat | [34, 66, 108] |
| Intracerebral hemorrhage | Rat | [109] |
| Spinal cord injury | Rat | [110] |
| Encephalomyelitis | Mouse | [111] |
| **Cardiovascular system** |  |  |
| Myocardical I/R injury | Rat | [94,112-115] |
| Cardiac cold I/R injury | Rat | [116,118] |
| Preservation of cardiac allografts | Rat | [71,119] |
| Left ventricular hypertrophy | Rat | [120] |
| Isoproterenol-induced myocardial infarction | Rat | [113] |
| Doxorubicin-induced myocardial injury | Rat | [121] |
| Cardiac arrest/resuscitation | Rat | [122] |
| Radiation-induced myocardial injury | Mouse | [123] |
| Postcardiac arrest syndrome | Rat | [124] |
| Atherosclerosis | Mouse | [125] |
| Carotid balloon injury | Rat | [126] |
| Cerebral vasospasm | Rat | [127] |
| **Digestive system** |  |  |
| Parasite-induced liver inflammation | Mouse | [11] |
| Concanavalin A-induced hepatitis | Mouse | [31] |
| Chronic hepatitis B | Human | [128] |
| Liver fibrogenesis | Mouse | [58] |
| Cirrhotic liver | Rat | [40] |
| Hepatic I/R injury | Mouse | [129] |
|  | Rat | [130] |
| Quality of life after radiotherapy for liver tumors | Human | [131] |
| Acetaminophen-induced hepatotoxicity | Mouse | [132] |
| Obstructive jaundice-induced liver injury | Rat | [45,133] |
| Nonalcoholic steatohepatitis and hepatocarcinogenesis | Mouse | [134] |
| Postoperative liver failure after major hepatectomy | Rat | [135] |
| Liver regeneration after partial hepatectomy | Rat | [39] |
| Acute hepatic injury in acute necrotizing pancreatitis | Rat | [136] |
| Non-alcoholic fatty liver disease | Rat | [137] |
| Intestinal I/R injury | Rat | [140-142] |
| Transplantation-induced intestinal injury | Rat | [27] |
| Preservation of the intestinal graft | Rat | [70] |
| Ulcerative colitis | Rat | [143] |
| Postoperative ileus | Human | [144] |
| Stress-induced gastric ulceration | Rat | [145] |
| Aspirin-induced gastric mucosal damage | Rat | [146] |
|  | Mouse | [147] |
| l-arginine-induced acute pancreatitis | Rat | [28] |
| Taurocholate-induced acute pancreatitis | Rat | [13] |
| I/R injury after pancreas transplantations | Rat | [148] |
| **Metabolism syndrome** |  |  |
| SHR.Cg-Leprcp/NDmcr rat - a metabolic syndrome rat model  Fructooligosaccharides diet-induced metabolic syndrome model | Rat  Rat | [153]  [151] |
| Potential metabolic syndrome | Human | [19,152] |
| High-fat diet-induced syndrome hamster model | Hamster | [154] |
| Obesity and diabetes | Mouse | [59,156] |
|  | Human | [155] |
| **Respiratory system** |  |  |
| Acute pulmonary I/R injury | Rat | [157] |
|  | Rabbit | [158] |
| Lung transplant-induced I/R injury | Rat | [32,159-160] |
| Septic-related lung injury | Rat | [37, 161] |
|  | Mouse | [162] |
| Hyperoxia-induced lung injury | Rat | [163-164] |
| Lipopolysaccharide-induced lung injury | Rat | [17] |
|  | Mouse | [14] |
| Smoke inhalation-induced lung injury | Rat | [165] |
| Paraquat-induced lung injury | Rat | [166] |
| Monocrotaline-induced lung injury | Rat | [167] |
| Extensive burn-induced lung injury | Rat | [168] |
| Cigarette smoking induced airway mucus production and epithelium damage | Rat | [169] |
| Asthma | Mouse | [46] |
| **Urinary system** |  |  |
| Renal I/R injury | Rat | [175-176] |
| Renal transplantation-induced renal cold I/R injury | Rat | [177] |
| Chronic allograft nephropathy | Rat | [178] |
| Acute renal injury after liver transplantation | Rat | [33] |
| Anti-cancer drug cisplatin-induced nephrotoxicity | Mouse | [60] |
| Ferric nitrilotriacetate-induced nephrotoxicity | Rat | [179] |
| Glucose and α,β-dicarbonyl compound-induced renal oxidative injury | Rat | [180] |
| Unilateral ureteral obstruction-induced renal injury | Rat | [181] |
| Spontaneous hypertensive-induced renal injury | Rat | [36] |
| Glycerol-induced acute renal injury | Rat | [43] |
| Septic shock-induced acute renal injury | Rat | [182] |
| Acute pancreatitis-induced renal injury | Rat | [183] |
| Severely burn-induced early acute renal injury  Interstitial cystitis/painful bladder syndrome | Rat  Human | [184]  [185] |
| **Reproduction system** |  |  |
| Radiation-induced testicular injury | Mouse | [188-189] |
| Gamma ray-induced testicular injury | Rat | [190] |
| Testicular I/R injury | Rat | [191] |
| Nicotine-induced testicular oxidative stress | Mouse | [193] |
| Erectile dysfunction in streptozotocin-induced diabetic model | Rat | [194] |
| Preeclampsia | Rat | [195] |
| Chemotherapy-induced ovarian injury | Rat | [196] |
| **Sensory system and skin diseases** |  |  |
| Retinal acute I/R injury | Rat | [72,198-199] |
| Oxygen-induced retinopathy | Mouse | [200] |
| Glutamate-induced retinal excitotoxic injury | Guinea pig | [201] |
| Light-induced retinal damage | Rat | [16] |
| Optic nerve crush | Rat | [202] |
| N-methyl-N-nitrosourea (MNU)-induced retinitis pigmentosa  Alkali burn-induced cornea injury | Rat  Mouse | [203]  [73] |
| Noise-induced temporary hearing loss | Guinea pig | [206-208] |
| Cisplatin-induced cochlear hair cells injury | Mouse | [204] |
| Antimycin A-induced vestibular hair cell injury | Mouse | [205] |
| Allergic rhinitis | Guinea pig | [209] |
| Local radiation-induced dermatitis | Rat | [210] |
| Ultraviolet(UV)-induced skin injury | Rat | [211] |
|  | Human | [212] |
| Acute erythemtous skin diseases | Human | [213] |
| Skin flap I/R injury | Rat | [214-215] |
| Psoriasis-associated skin lesions | Human | [216] |
| Post herpetic neuralgia | Rat | [217] |
| **Tissue dysfunction** |  |  |
| Oxidative stress in cultured chondrocytes | Pig and rat | [218] |
| Rheumatoid arthritis | Human | [219] |
| Bone loss induced by microgravity | Rat | [220] |
| Periodontitis | Rat | [221-222] |
| Oxidative stress injury in cultured human gingival fibroblasts  Steroid-induced osteonecrosis | Human  Rabbit | [223]  [224-225] |
| Acute graft-versus-host disease | Mouse | [226-227] |
| Sepsis | Rat | [229-230] |
|  | Mouse | [228] |
|  | Rat and Mouse | [231] |
| Aplastic anemia | Mouse | [232] |
| Blood alkalinity | Human | [233-234] |
| Collagen-induced platelet aggregation | Human and Rat | [235] |
| Serum anti-oxidative function | Horse | [236] |
| Mitochondrial and inflammatory myopathies | Human | [237] |
| Duchenne muscular dystrophy | Mouse | [238] |
| Acute exercise-induce muscle fatigue | Human | [239] |
| Balloon injury-induced neointimal hyperplasia | Rat | [49] |
| Rhabdomyolysis | Rat | [43] |
| Skeletal muscle I/R injury | Rat | [240] |
| Myocardial muscle I/R injury | Rat | [112,241] |
| **Cancer and complications** |  |  |
| Skin squamous cell carcinoma | Mouse | [10] |
| Cultured human tongue carcinoma cells | Human | [245] |
| Radiation-induced thymic lymphoma | Mouse | [246] |
| Nonalcoholic steatohepatitis and accompanying hepatocarcinogenesis | Mouse | [134] |
| Nephrotoxicity and early tumor promotional events | Rat | [179] |
| Liver tumors | Human | [131] |
| Cisplatin-induced nephrotoxicity | Rat | [247] |
|  | Mouse | [60] |
| Doxorubicin-induced cardiac and hepatic injury | Rat | [121] |
